# Fixation times on directed graphs

**DOI:** 10.1371/journal.pcbi.1012299

**Published:** 2024-07-18

**Authors:** David A. Brewster, Martin A. Nowak, Josef Tkadlec

**Affiliations:** 1 John A. Paulson School of Engineering and Applied Sciences, Harvard University, Cambridge, Massachusetts, United States of America; 2 Department of Mathematics, Harvard University, Cambridge, Massachusetts, United States of America; 3 Department of Organismic and Evolutionary Biology, Harvard University, Cambridge, Massachusetts, United States of America; 4 Computer Science Institute, Charles University, Prague, Czech Republic; Universitat zu Koln, GERMANY

## Abstract

Computing the rate of evolution in spatially structured populations is difficult. A key quantity is the fixation time of a single mutant with relative reproduction rate *r* which invades a population of residents. We say that the fixation time is “fast” if it is at most a polynomial function in terms of the population size *N*. Here we study fixation times of advantageous mutants (*r* > 1) and neutral mutants (*r* = 1) on *directed* graphs, which are those graphs that have at least some one-way connections. We obtain three main results. First, we prove that for any directed graph the fixation time is fast, provided that *r* is sufficiently large. Second, we construct an efficient algorithm that gives an upper bound for the fixation time for any graph and any *r* ≥ 1. Third, we identify a broad class of directed graphs with fast fixation times for any *r* ≥ 1. This class includes previously studied amplifiers of selection, such as Superstars and Metafunnels. We also show that on some graphs the fixation time is not a monotonically declining function of *r*; in particular, neutral fixation can occur faster than fixation for small selective advantages.

## Author summary

Populations of reproducing individuals evolve by accumulating mutations. When a single individual acquires a mutation, it takes some time until the mutation spreads across the population. Depending on the structure of the population, this so-called fixation time could be relatively short or exceedingly long. The structure of biological populations can be represented by graphs. The vertices of the graph denote individuals and the edges indicate possible reproductive events. Directed graphs are defined as those graphs that have some one-way edges. It is known that very long fixation times can arise for certain directed graphs. In this work, we consider a standard process of evolutionary dynamics. We show that for many directed graphs the fixation time is short. We show that fixation time is always short in the ecological scenario describing the spread of an invasive species in an ecosystem. We also show that while advantageous mutations generally fixate faster than neutral mutations, there are interesting exceptions. Finally, we provide concrete bounds for fixation time on any population structure. Those bounds serve as guarantees for simulation-based and computational explorations of the space of all directed graphs.

## Introduction

Evolution is a stochastic process that acts on populations of reproducing individuals. Two main driving forces of evolutionary dynamics are mutation and selection [1–3]. Mutation generates new variants and selection prunes them. When new mutations are sufficiently rare, the evolutionary dynamics are characterized by the fate of a single new mutant. The mutant can either take over the whole population or become extinct. Even when the mutation grants its bearer a relative fitness advantage *r* ≥ 1, it might still go extinct due to random fluctuations [4]. Two key parameters that quantify the fate of the newly occurring mutation are the fixation probability and the fixation time [5, 6]. Here we study the effect of spatial structure on those quantities.

Spatial models have a long history of investigation in ecology [7–13], population dynamics [4], population genetics [6, 14–19], evolutionary game theory [20–24], infection dynamics [25–28] and cancer evolution [29, 30]. The classical investigation in population genetics includes the debate between Fisher and Wright [15] and hybrid zones [31]. A biological population can be structured in the sense of geographical distribution, age structure, or specific interaction patterns. Human populations structure is often studied in terms of social networks [32].

Spatial structure has profound effects on both the fixation probability and the fixation time. Those effects are studied within the framework of Evolutionary Graph Theory [33–36]. There, individuals are represented as nodes of a graph (network). The edges (connections) of the graph represent the migration patterns of offspring. The edges can be one-way or two-way. Graphs can represent the well-mixed population, spatial lattices, island sub-populations, or arbitrary complex spatial structures. Those directed graphs can arise by the flow from upstream to downstream demes in meta-populations, by cellular differentiation in somatic evolution, or in age structured populations. Also in human social networks many interactions are one way—such as from influencer to follower or from teacher to learner.

Previous research investigated population structures with various effects on fixation probability and time [37–41]. For example, isothermal graphs have both the same fixation probability and the same distribution of mutant population size changes over time as the well-mixed population [33, 40]. Suppressors of selection reduce the fixation probability of advantageous mutants [42], and amplifiers of selection enhance the fixation probability of advantageous mutants [43]. Amplifiers are population structures that could potentially accelerate the evolutionary search [44]. Known classes of amplifiers include families such as Stars [45–47], Comets [48], Superstars [49, 50], or Megastars [51].

Interestingly, the amplification typically comes at a cost of increasing the fixation time [52], sometimes substantially [53]. This is problematic, since when fixation times are extremely long, fixation is not a relevant event any more, and thus the fixation probability alone is not the most representative quantity [54, 55]. It is therefore paramount to understand how the population structure affects the fixation time and, in particular, what are the population structures for which the fixation time is “reasonably fast.”

Borrowing standard concepts from computer science [56], in this work we say that fixation time is *fast* if the fixation time is (at most) polynomial in terms of the population size *N*. Otherwise we say that the fixation time is *long*, and the corresponding population structure is *slow*. Two important known results are: (i) for all *undirected* graphs the fixation time is fast [57, 58]; and (ii) if some edges are one-way (if the graph is directed), then the fixation time can be exponentially long [59]. The latter result has an important negative consequence: when the fixation time is exponentially long, we know no tool to efficiently simulate the process. Therefore, computing or approximating any relevant quantities for realistic population sizes is in practice infeasible.

In this work, we present three positive results that concern fixation times on directed graphs (where some or all edges are one-way). First, we prove that for any directed graph the fixation time is fast, provided that the mutant fitness advantage *r* is sufficiently large. Second, we devise an efficient algorithm that gives an upper bound on the fixation time, for any graph and any *r* ≥ 1. The bound can be used to estimate how long one needs to run the simulations until they terminate. Third, we identify a broad class of directed graphs for which the fixation times are fast for any *r* ≥ 1. This class includes many previously studied amplifiers of selection, such as Superstars and Metafunnels. To conclude, we discuss important algorithmic consequences that enable efficient computational exploration of various properties of directed graphs.

## Model

In this section we define the notions we use later, such as the population structure (represented by a graph), the evolutionary dynamics (Moran Birth-death process), and the key quantities (fixation probability and fixation time).

### Population structure

The spatial population structure is represented by a graph (network) *G* = (*V*, *E*), where *V* is the set of *N* nodes (vertices) labeled *v*_1_, …, *v*_*N*_ and *E* is the set of directed one-way edges (links) connecting pairs of different nodes. A two-way connection between nodes *u* and *v* is represented by two one-way edges *u* → *v* and *v* → *u*. We assume that the graph is strongly connected. For any node *v*, the number of edges incoming to *v* is called the indegree (denoted deg^−^(*v*)), and the number of outgoing edges is called the outdegree (denoted deg^+^(*v*)). When the two numbers coincide, we call them the degree (denoted deg(*v*)).

### Graph classes

We say that a graph is *undirected* if for every edge *u* → *v* there is also an edge *v* → *u* in the opposite direction. Otherwise we say that a graph is *directed*. We say that a graph is *regular* if all nodes have the same degree, that is, there exists a number *d* such that deg^−^(*v*) = deg^+^(*v*) = *d* for all nodes *v* ∈ *V*. We say that a graph *G* is *Eulerian* (also known as a circulation) if deg^−^(*v*) = deg^+^(*v*) for each node *v*. Finally, in this work we say that a graph is *balanced* if an equality

$$\frac{1}{{\text{deg}}^{+}\left(v\right)}\cdot \sum_{w\in V:v\to w\in E}\frac{1}{{\text{deg}}^{-}\left(w\right)}=\frac{1}{{\text{deg}}^{-}\left(v\right)}\cdot \sum_{u\in V:u\to v\in E}\frac{1}{{\text{deg}}^{+}\left(u\right)}$$

holds for all nodes *v*. Here the left-hand side represents the average indegree of the successors of *v*, whereas the right-hand side represents the average outdegree of the predecessors of *v*. It is straightforward to check that the class of balanced graphs includes the regular graphs and the undirected graphs, as well as other graph classes such as Superstars or Metafunnels [33], see S1 Appendix. Below we will prove that the fixation times on all balanced graphs are fast for any *r* ≥ 1.

### Moran Bd process

To model the evolutionary dynamics we consider the standard Moran Birth-death process. Each node of the graph is occupied by a single individual. Initially, some individuals are wild-type residents with normalized fitness equal to 1, and some individuals are mutants with relative fitness advantage *r* ≥ 1. Given a graph *G* and a relative fitness advantage *r* ≥ 1, Moran Birth-death process is a discrete-time stochastic process, where in each step:

1. First (Birth), we select an individual with probability proportional to its fitness. Suppose we selected node *u*.
2. Second (death), we select an outgoing neighbor of *u* uniformly at random. Suppose we selected node *v*.
3. Finally (update), we replace the individual at node *v* by a copy of individual at node *u*.

At each time-step, the current *configuration* is the subset of nodes occupied by mutants. Since the graph is strongly connected, almost surely we eventually obtain a configuration where either all nodes are mutants (we say that mutants *fixed*), or all nodes are residents (we say that mutants *went extinct*) [57].

### Fixation probability and fixation time

The key quantities that we consider in this work are fixation probability and fixation time.

Given a graph *G*, a mutant fitness advantage *r* ≥ 1, and an initial configuration *S* ⊆ *V* of nodes occupied by mutants, the *fixation probability* fp_*r*_(*G*, *S*) is the probability that starting from *S*, the mutants eventually fix (as opposed to going extinct). Morever, we define an auxiliary quantity fp_min_ that turns out to be useful later in our results. Formally, given a graph *G* and *r* = 1, for *i* = 1, …, *N* denote by fp^(*i*)^(*G*) = fp_*r*=1_(*G*, {*v*_*i*_}) the fixation probability of a single neutral mutant who initially appears at node *v*_*i*_. We define fp_min_(*G*) = min_*i*_ fp^(*i*)^(*G*) to be the smallest of those *N* fixation probabilities.

To measure the duration of the process until fixation (or extinction) occurs, different notions are used. The *(expected) absorption time* AT_*r*_(*G*, *S*) is the expected number of steps of the Moran Birth-death process until the process terminates, regardless of what is the outcome (mutant fixation or extinction). In contrast, *(expected) fixation time* T_*r*_(*G*, *S*) is the expected number of steps averaged over only those evolutionary trajectories that terminate with mutant fixation. Similarly, one can define the *(expected) extinction time* ExtT_*r*_(*G*, *S*) averaging over only those trajectories that terminate with the mutant going extinct. By linearity of expectation, the three quantities are related as AT_*r*_(*G*, *S*) = fp_*r*_(*G*, *S*) ⋅ T_*r*_(*G*, *S*) + (1 − fp_*r*_(*G*, *S*)) ⋅ ExtT_*r*_(*G*, *S*). Note that in this work, absorption time, fixation time, and extinction time are mean times to absorption, making them scalar values rather than random variables. Information about the random variable can be recovered from its expectation using concentration bounds such as Markov’s inequality [57]. Our objective in this work is to provide upper bounds on the absorption time and on the fixation time. To that end, given a graph *G* and a mutant fitness advantage *r* ≥ 1, let T_*r*_(*G*) = max_*S*⊆*V*,*S*≠∅_ T_*r*_(*G*, *S*) be the largest fixation time among all possible initial configurations. In the limit of strong selection *r* → ∞ we also define T_∞_(*G*) = lim_*r*→∞_*T*_*r*_(*G*). This regime is called the ecological scenario [60] and corresponds to new invasive species populating an existing ecosystem.

### Asymptotic notation

We say a function *f*(*N*) is (at most) *polynomial* if there exists a positive constant *c* such that *f*(*N*) ≤ *N*^*c*^ for all large enough *N*. Examples of polynomial functions are $f\left(N\right)=\frac{1}{2}N\left(N+1\right)$ and *f*_2_(*N*) = 10 ⋅ *N* log *N*, whereas functions such as *g*(*N*) = 1.1^*N*^ and $g_{2}\left(N\right)=2^{\sqrt{N}}$ are not polynomial, since they grow too quickly. In computer science, problems that can be solved using polynomially many elementary computations are considered tractable. In alignment with that, given a population structure *G* with *N* nodes, we say that fixation time is *fast* if it is at most polynomial in terms of the population size *N*.

## Results

We present three main types of results.

### Fixation time is fast when selection advantage is strong enough

As our first main result, we prove that the fixation time on any directed graph is fast, provided that the mutant fitness advantage *r* is large enough.

**Fig 1:**
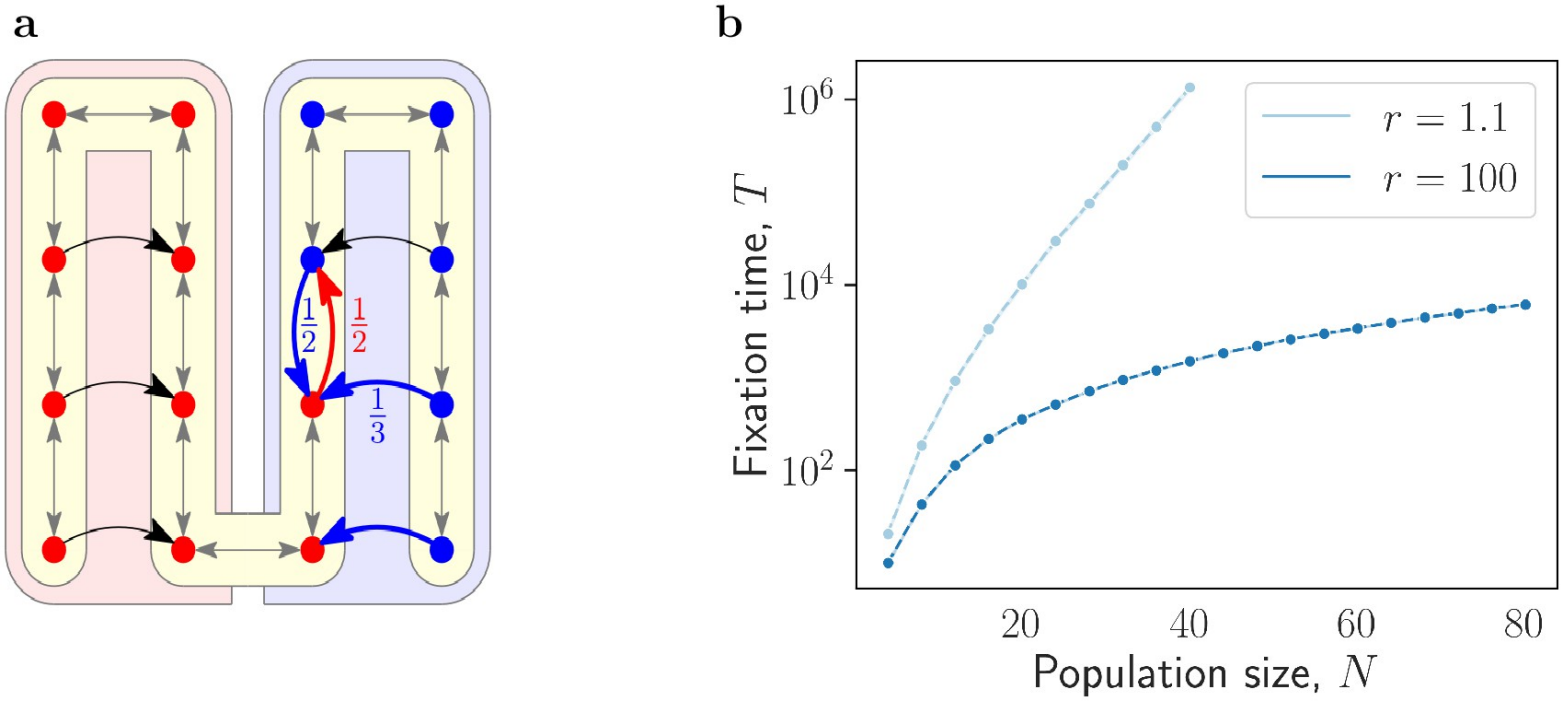
Long and fast fixation times on a four-column graph *G*_*N*_. **a**, For *N* = 4*k*, a graph *G*_*N*_ consists of four columns of *k* nodes each (here *k* = 4 and *N* = 16). The grey edges within the yellow region are two-way, the black edges going from the side columns to the corresponding vertices of the middle column are one-way. The fractions shown highlight the probability that a step of the Birth-death occurs between the start and end vertex on the corresponding edge given the individual at the start vertex is selected for birth. Initially mutants occupy the left half and residents occupy the right half. As mutants (red nodes) spread upward through the third column, they can propagate along only one edge (red), whereas residents (blue nodes) fight back along multiple edges (blue). **b**, The timescale to fixation crucially depends on the mutant fitness advantage *r*. When *r* = 1.1 and the initial configuration *S* is all of the nodes on the left half, the fixation time T_*r*_(*G*_*N*_, *S*) is exponential in *N*, whereas when *r* = 100 it is polynomial. Each data point is an average over at least 10^3^ simulations.

As an illustration, for every *N* = 4*k* we consider a graph *G*_*N*_ depicted in Fig 1A. It consists of four columns of *k* nodes each. The grey edges within the yellow region are two-way. The black one-way edges point from the side columns to the middle columns. When mutants initially occupy the left part of the graph, the only way forward for them is to progress upward through the third column. But while there, mutants are under an increased pressure due to the resident nodes in the rightmost column. The same applies to residents. They can only make progress by climbing upward through the second column, but there they are under pressure due to mutants in the leftmost column. As a consequence, the fixation time crucially depends on *r*. When *r* = 1.1, Fig 1B shows that the fixation time scales exponentially in *N* (that is, it is long). In contrast, in the limit of large *r* the fixation time is less than *N*^2^, that is, it is fast.

In general, we can prove the following result about an arbitrary population structure.

**Theorem 1**. *Let G*_*N*_ *be a strongly connected graph on N nodes. Suppose that r* ≥ *N*^2^. *Then* AT_*r*_(*G*_*N*_) ≤ 2*N*^3^ *and* T_*r*_(*G*_*N*_) ≤ 3*N*^3^.

Theorem 1 implies that while the fixation time on certain graphs can be long for some values of *r*, this effect is inevitably transient, and the fixation time becomes fast once *r* exceeds a certain threshold. The intuition behind the proof is that if *r* is large enough, the size of the mutant subpopulation is always more likely to increase than to decrease, regardless of which nodes are currently occupied by mutants. Thus, the evolutionary process can be mapped to a random walk with a constant positive bias. It is known that such biased random walks absorb polynomially quickly. See S1 Appendix for details.

An attractive feature of Theorem 1 is that it applies to all directed graphs. An obvious limitation is that the condition *r* ≥ *N*^2^ is unrealistic, except possibly for the regime *r* → ∞ that has been studied under the name ecological scenario [60]. Therefore, as our second result, we considerably relax this condition for graphs with certain structural features. A directed graph is said to be Eulerian (also called a *circulation*) if each node has the same indegree as outdegree. In that case, we refer to the number deg^−^(*v*) = deg^+^(*v*) simply as a *degree* of node *v*.

**Theorem 2**. *Let G*_*N*_ *be a strongly connected Eulerian graph on N nodes with smallest degree δ and largest degree* Δ. *Suppose that* $r\geq \frac{\Delta }{\delta }\cdot \left(1+\varepsilon \right)$ *for some ε* > 0. *Then* ${\text{AT}}_{r}\left(G_{N}\right)\leq \frac{2+\varepsilon }{\varepsilon }\cdot N^{3}$ *and* ${\text{T}}_{r}\left(G_{N}\right)\leq \frac{\left(1+\varepsilon )(2+\varepsilon \right)}{\varepsilon^{2}}\cdot N^{3}$.

To illustrate Theorem 2 we point out two special cases (for the full proof see S1 Appendix).

First, consider any regular graph *G*_*N*_, that is, a graph where all nodes have the same indegree and outdegree equal to *d*. Then, the graph is Eulerian and we have *d* = Δ = *δ*, and thus Theorem 2 implies that T_*r*_(*G*_*N*_) is at most a polynomial in *N* and *d* for any *r* ≥ 1. In other words, fixation time on any regular graph is fast for any *r* ≥ 1 (we note that this result is known [59]).

Second, consider an Eulerian graph that is “close to being regular”, in the sense that each node has degree either 4 or 5. An example of such a graph is a square lattice with several additional long-range connections. Then, Theorem 2 implies that the fixation time is fast for every *r* > 5/4 = 1.25.

### Fixation time for small selective advantage

**Fig 2:**
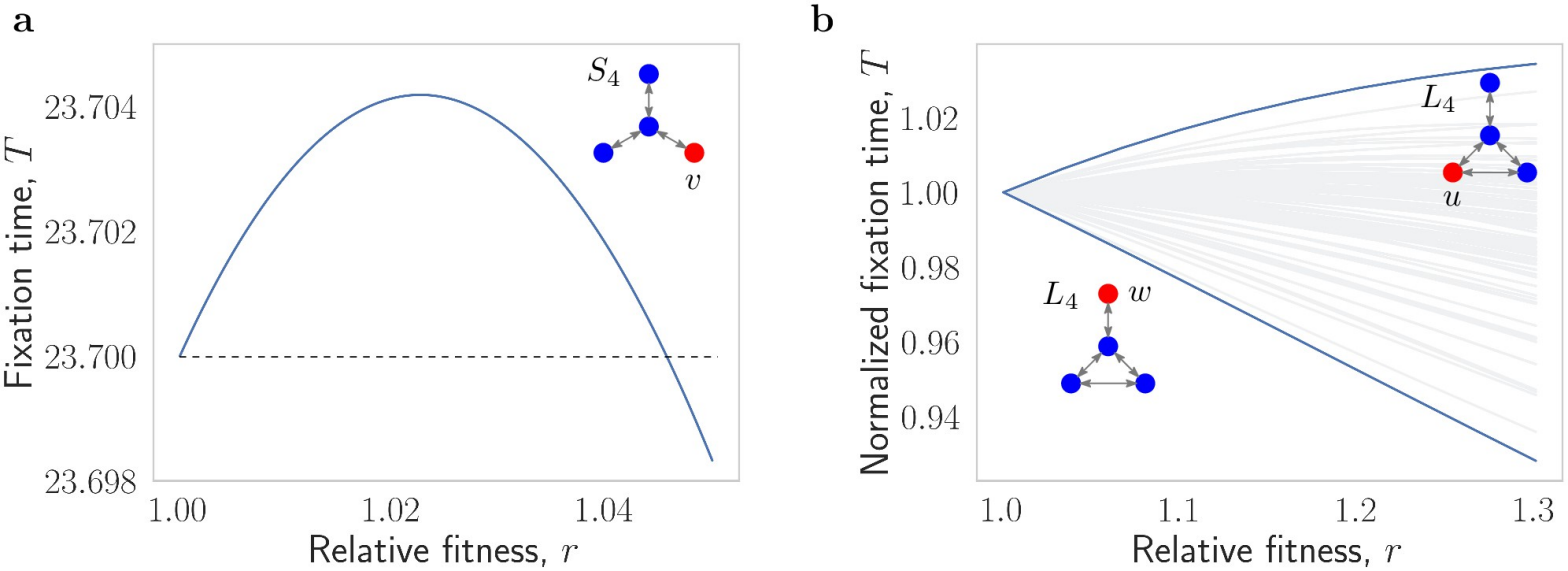
Fixation time is not monotone in *r*. **a**, In an (undirected) star graph *S*_4_ on 4 nodes, one node (center) is connected to three leaf nodes by two-way edges. When the initial mutant appears at a leaf *v*, the fixation time T_*r*_(*S*_4_, {*v*}) increases as *r* increases from *r* = 1 to roughly *r* = 1.023. Then it starts to decrease. **b**, Normalized fixation time T_*r*_(*G*, {*v*})/T_*r* = 1_(*G*, {*v*}) as a function of *r* ∈ [1, 1.3], for all 83 strongly connected graphs *G* with 4 nodes, and all four possible mutant starting nodes *v*. As *r* increases, the fixation time goes up for 182 of the 4 ⋅ 83 = 332 possible initial conditions. The increase is most pronounced for the so-called *lollipop* graph *L*_4_ and a starting node *u*. In contrast, for the same lollipop graph and a different starting node *w*, the fixation time decreases the fastest.

The above results show that for any fixed graph *G*, the fixation time is fast when *r* is sufficiently large. It is natural to hope that perhaps for any fixed graph *G* the fixation time is a monotonically decreasing function of *r* for *r* ≥ 1. However, this is not the case, as shown in Fig 2.

Briefly speaking, the effect responsible for the increase in fixation time when *r* = 1 + *ε* is that by increasing the mutant fitness advantage, certain evolutionary trajectories that used to lead to mutant extinction instead lead to mutant fixation. Since those “newly fixating” trajectories might generally take relatively long to fix, the average length of the fixating trajectories can go up. Similarly, the absorption time can also go up as we increase *r*. Those findings are in alignment with the stochastic slowdown phenomenon [61].

Despite the lack of monotonicity, we can show that the fixation time cannot go up too much as we increase *r*. Recall that fp_min_(*G*_*N*_) = min{fp_*r*=1_(*G*_*N*_, {v}) ∣ *v* ∈ *G*_*N*_} denotes the fixation probability under neutral drift (*r* = 1), when the initial mutant appears at a node *v* with the smallest fixation probability. Note that for any graph with *N* nodes we have fp_min_(*G*_*N*_) ≤ 1/N, but fp_min_(*G*_*N*_) could in general be substantially smaller than 1/*N*. Finally, the quantity fp_min_(*G*_*N*_) can be computed efficiently by solving a linear system of *N* equations [35, 39, 62, 63]; see S1 Appendix for additional details.

We can now state our second main result.

**Theorem 3**. *Let G*_*N*_ *be a strongly connected graph on N vertices and let r* ≥ 1. *Then* ${\text{T}}_{r}\left(G_{N}\right)\leq \frac{N^{6}}{{\left({\text{fp}}_{\text{min}}\left(G_{N}\right)\right)}^{4}}.$

We note that Theorem 3 yields an efficiently computable upper bound on T_*r*_(*G*_*N*_). In the next section we elaborate on the computational consequences of this result. In the rest of this section, we give a brief intuition behind the proof of Theorem 3.

The proof relies on two ingredients. First, instead of considering the process with mutant fitness advantage *r*, we consider the neutral process that corresponds to *r* = 1. There, using a martingale argument we show that the fixation time T_*r*=1_(*G*_*N*_) can be bounded from above in terms of the quantity fp_min_. The intuition is that as long as all fixation probabilities are non-negligible, all active steps of the stochastic process have substantial magnitude either towards fixation or towards extinction. As a consequence, we are able to argue that either fixation or extinction will occur after not too many steps. All in all, this yields an upper bound on fixation time T_*r*=1_(*G*_*N*_) of the neutral process in terms of the quantity fp_min_. See S1 Appendix for details.

As our second ingredient, we translate the bound on T_*r*=1_(*G*_*N*_) into a bound on T_*r*_(*G*_*N*_) for any *r* ≥ 1. We note that, as indicated in Fig 2, for a fixed graph *G*_*N*_ the fixation time is in general not a monotonically decreasing function of *r*. Nevertheless, the continuous versions of two processes can be coupled in a certain specific way, which allows us to argue that while T_*r*_(*G*_*N*_) can be somewhat larger than T_*r*=1_(*G*_*N*_), it cannot be substantially larger. In this step, we again use the quantity fp_min_. See S1 Appendix for details.

### Fixation time is fast when the graph is balanced

As noted above, Theorem 3 provides an upper bound on the fixation time for any graph *G*_*N*_ and any mutant fitness advantage *r* ≥ 1, in terms of the quantity fp_min_(*G*_*N*_). We have 0 ≤ fp_min_(*G*_*N*_) ≤ 1/*N*. When the quantity fp_min_(*G*_*N*_) is exponentially small, the upper bound from Theorem 3 becomes exponentially large, and thus not particularly interesting. However, for many graphs the quantity fp_min_(*G*_*N*_) turns out to be much larger, namely inversely proportional to a polynomial in *N*. In those cases, Theorem 3 implies that the fixation time T_*r*_(*G*_*N*_) is fast for any *r* ≥ 1.

**Fig 3:**
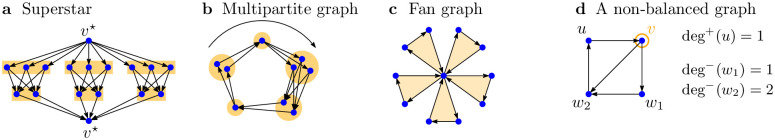
Types of balanced graphs. The class of balanced graphs includes the following families of graphs studied in the context of Moran process in the existing literature. **a**, Superstars [33] are the first proposed strong amplifiers of selection. **b**, Complete multipartite graphs [65] are a rare example of high-dimensional graphs for which the fixation probability of advantageous mutants can be expressed using an explicit formula. **c**, A certain form of Fan graphs [66] (with weighted and undirected edges) constitutes the strongest currently known amplifiers of selection under death-Birth updating. Theorem 4 implies that the fixation time on all those graphs is fast for all *r* ≥ 1. **d**, Not all graphs are balanced. For example, here for the highlighted node *v* the left hand side is 1, whereas the right-hand side is $\frac{1}{2}\left(1/1+1/2\right)=0.75\neq 1$.

In particular, as our third main result we prove that this occurs for a broad class of graphs which we call balanced graphs. Formally, we say that a graph *G*_*N*_ is *balanced* if an equality

$$\frac{1}{{\text{deg}}^{-}\left(v\right)}\cdot \sum_{u:u\to v\in E}\frac{1}{{\text{deg}}^{+}\left(u\right)}=\frac{1}{{\text{deg}}^{+}\left(v\right)}\cdot \sum_{w:v\to w\in E}\frac{1}{{\text{deg}}^{-}\left(w\right)}$$

holds for all nodes *v*. Here the left-hand side represents the average outdegree of the predecessors of *v*, whereas the right-hand side represents the average indegree of the successors of *v*. We note that the family of balanced graphs includes many families of graphs studied in the context of Moran process in the existing literature, such as the undirected graphs [57], regular (possibly directed) graphs [59], Superstars and Metafunnels [33], Megastars [51, 64], cyclic complete multipartite graphs [65], or directed fans [66], see Fig 3.

We have the following theorem.

**Theorem 4**. *Let G*_*N*_ *be a balanced strongly connected graph. Then*:

1.
${\text{fp}}_{r=1}\left(G_{N},u\right)=\frac{1/{\text{deg}}^{-}\left(u\right)}{\sum_{v\in V}1/{\text{deg}}^{-}\left(v\right)}\geq 1/N^{2}$ *for any node u*.
2. T_*r*_(*G*_*N*_) ≤ *N*^14^ *for any* *r* ≥ 1.

Theorem 4 implies that the fixation time on all balanced graphs is fast for all *r* ≥ 1. Similarly, we can prove that it is fast for Megastars [51] assuming *r* ≥ 1 (see S1 Appendix).

The proof of the first part of Theorem 4 relies on the fact that in the neutral case *r* = 1 the fixation probability is additive. This allows us to reduce the size of the linear system that describes the underlying Markov chain from 2^*N*^ equations to *N* equations. For balanced graphs, this system takes a special form that admits an explicit solution. The second part then follows directly from Theorem 3. See S1 Appendix for details.

We note that the second part of Theorem 3 has an important computational consequence. Since the fixation time on any balanced graph is bounded from above for any *r* ≥ 1, individual-based simulations of the evolutionary process are guaranteed to terminate quickly with high probability [57]. Any relevant quantities of interest, such as the fixation probability of the mutant with *r* ≥ 1, can thus be efficiently approximated to arbitrary precision. In particular, Theorem 3 yields a fully-polynomial randomized approximation scheme (FPRAS) for the fixation probability on balanced graphs with any *r* ≥ 1.

**Theorem 5**. *There is a FPRAS for fixation probability on balanced graphs for any r* ≥ 1.

We note that Theorem 5 applies also to any (not necessarily balanced) graph *G*_*N*_, provided that the quantity fp_min_(*G*_*N*_) is inversely proportional to a polynomial. This is the case for instance for Megastars. See S1 Appendix for details. Moreover, when fp_min_(*G*_*N*_) is smaller than that, Theorem 3 still gives an explicit, efficiently computable upper bound on the fixation time that can be used to bound the running time of any individual-based simulations.

### Computational experiments

**Fig 4:**
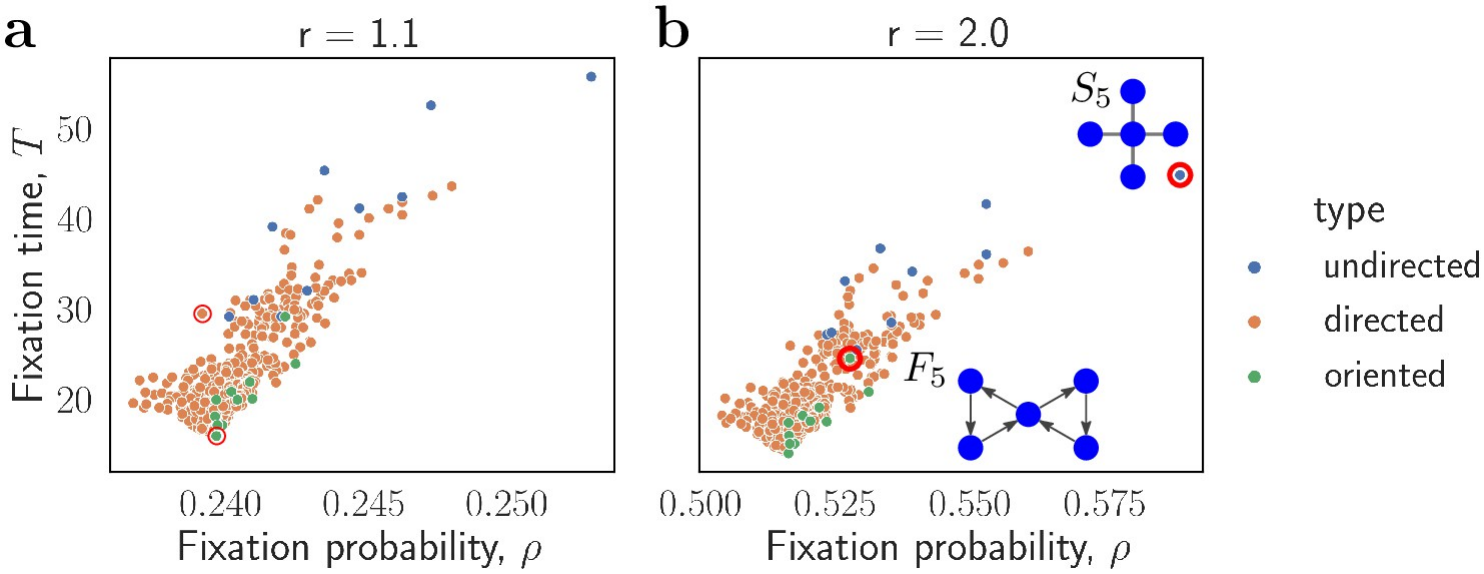
Fixation time and fixation probability of a single mutant under uniform initialization for all 5048 graphs with *N* = 5 nodes, for **a**, *r* = 1.1 and **b**, *r* = 2. Each graph is represented as a colored dot. The undirected graphs (with all edges two-way) are labeled in blue. The oriented graphs (with no edges two-way) are labeled in green. All other directed graphs are labeled in orange. The slowest graph is the (undirected) Star graph *S*_5_. Among the oriented graphs, the slowest graph is the Fan graph *F*_5_.

Finally, to further illustrate the scope of our results we run several computational experiments on graphs with small population size *N*. We use nauty [67] to perform such enumerations. Since already for *N* = 6 there are more than one million non-isomorphic strongly connected directed graphs, we consider *N* = 5. For each of the 5048 graphs with *N* = 5 we compute the fixation time and the fixation probability under uniform initialization by solving the underlying Markov chain using numerical methods, see Fig 4.

The slowest graph is the (undirected) Star graph. Note that when *N* is large the fixation time on a Star graph is known to be proportional to roughly *N*^2^ [52].

**Fig 5:**
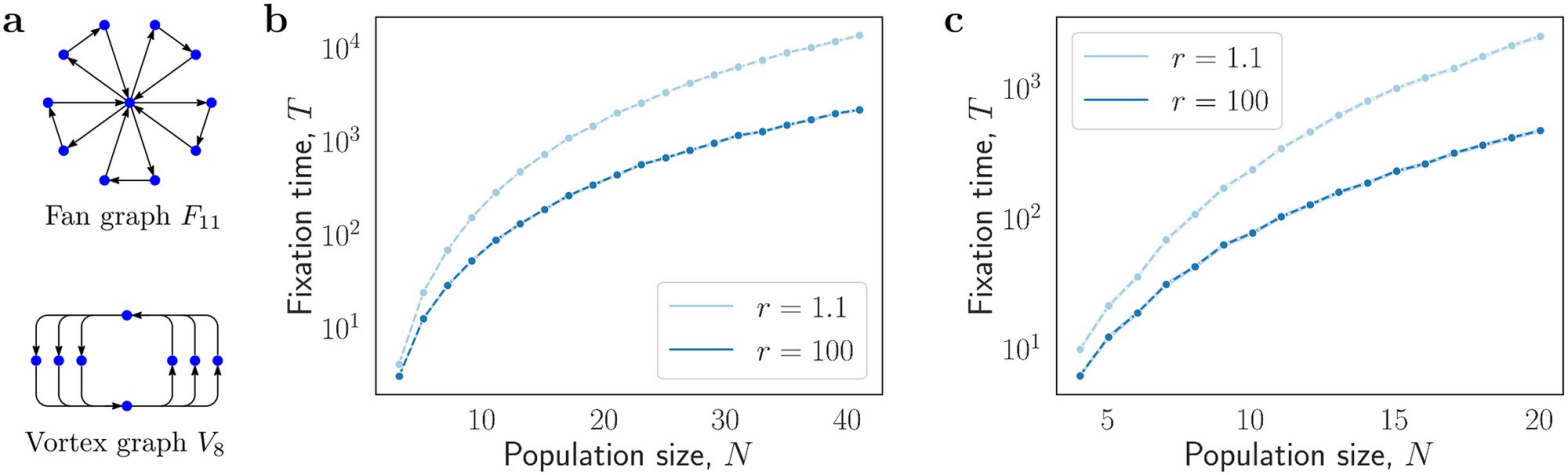
Fixation time on slow oriented graphs. **a**, The Fan graph with *k* blades has *N* = 2*k* + 1 nodes and 3*k* one-way edges (here *k* = 5 which yields *N* = 11). The Vortex graph with batch size *k* has *N* = 2*k* + 2 nodes and 4*k* edges (here *k* = 3 which yields *N* = 8). **b-c**, For both the Fan graphs and the Vortex graphs the fixation time scales roughly as *N*^2^, both for *r* = 1.1 and *r* = 100. (Each data point is an average over 1000 simulations).

Among the oriented graphs, the slowest are variants of either a fan graph *F*_*N*_, or a vortex graph *V*_*N*_. Since both the fan graphs and the vortex graphs belong to the class of balanced graphs, the fixation time on those graphs is fast for any population size *N* and any mutant fitness advantage *r* ≥ 1 due to Theorem 4 (see Fig 5 for empirical support). The fixation time appears to be proportional to roughly *N*^2^ (see S1 Appendix).

Together, those results suggest that even though directed graphs with exponentially long fixation times do exist, in practice most small directed graphs reach fixation reasonably quickly.

## Discussion

A decade ago, a foundational work by Diaz et. al. showed that fixation time on any undirected population structure is fast [57]. This result enabled extensive computational exploration of the landscape of all undirected graphs that later lead to several inspiring research outputs [20, 37, 54, 68, 69]. It is our hope that by enabling computational exploration of population structures with some (or all) one-way connections, this work will serve the same purpose.

Studying the evolutionary dynamics in spatially structured populations is notoriously hard. In this work we consider one of the simplest possible dynamics, namely the classic Moran Birth-death process, and we analyze the fixation time of a newly occurring mutant. When the fixation time is exponentially long, the process is expensive to simulate, and moreover various commonly studied quantities such as fixation probability are largely irrelevant. It is thus paramount to delineate settings in which the fixation time is “relatively fast”, as opposed to being “exceedingly long.”

It is known [57] that the fixation time is fast, provided that all interactions among the individuals are two-way. However, many relevant population structures include one-way interactions. In meta-population dynamics, an upstream deme could seed a downstream deme. In somatic evolution, cellular differentiation could be irreversible. In an age structured population there is the one way arrow of time.

In this work, we therefore consider spatial structures in which some (or all) interactions are one-way. It is known that on such structures the fixation time can be exceedingly long [59]. Nevertheless, here we present three results which indicate that fixation times on spatial structures with one-way connections are often fast.

First, we prove that on any population structure the fixation time is fast, provided that the mutant fitness advantage *r* exceeds a certain threshold value *r*^⋆^ (see Theorem 1). In the special case when the population structure is represented by a regular graph, the threshold value simplifies to *r*^⋆^ = 1, and we recover a known result that the fixation time on regular graphs is short for all *r* ≥ 1 [59]. As another corollary, for any Eulerian graph whose degrees are sandwiched between *δ* and Δ we can set *r*^⋆^ = Δ/*δ* (see Theorem 2).

Second, somewhat counter-intuitively we show that fixation time sometimes goes up as we increase *r*. That is, on certain spatial structures fixation of a neutral mutant occurs faster than fixation of a mutant with a small selective advantage. This effect is called stochastic slowdown [61]. We show that the magnitude of the slowdown can be bounded. In particular, in the spirit of parametrized complexity [70, 71], given a graph structure *G*_*N*_ we define a certain efficiently computable quantity fp_min_(*G*_*N*_), and we bound the fixation time for any *r* ≥ 1 from above using fp_min_(*G*_*N*_) and *N* (see Theorem 3). This has important consequences for performing individual-based simulations that typically run the process several times and report an empirical average. The limitation of naive individual-based simulations is that, a priori, it is not clear how much time will be needed until the simulations converge, and deciding to stop the simulations mid-way may bias the empirical average by over-representing the evolutionary trajectories that quickly go extinct. Using Theorem 3, this limitation can be circumvented by first efficiently computing an upper bound on the expected fixation time without having to simulate the process even once. We stress that this computational approach works for all spatial structures, balanced or not, and hence it expands our methodological toolkit for computational treatment of the role of spatial structure in evolution.

Third, we identify a class of population structures for which fixation times are fast for any *r* ≥ 1. This class is surprisingly broad. To start with, it includes several families of graph that had been studied in the context of Evolutionary Graph Theory earlier, such as Superstars and Metafunnels [33], or directed Fans [66]. Furthermore, the class also includes several other graph families of general interest, such as book graphs or cyclic complete multipartite graphs [65]. Similarly, we prove that the fixation times on Megastars [51] are fast for all *r* ≥ 1 too.

While the focus of this work is to identify regimes and population structures that lead to fast fixation times, population structures with long fixation times may also be desirable, e.g. in conservation ecology to maintain high levels of ecological diversity [72–76]. Our results imply that spatial structures that support coexistence of two competing types on exponential time-scales all have a common feature. Namely, there must exist a starting node such that in the neutral regime (*r* = 1), the fixation probability of a single mutant who initially appears at that node is exponentially small. In other words, spatial structures in which a neutral mutant has a non-negligible chance of fixating, no matter where it appears, never support coexistence on long time-scales.

We note that throughout this work we considered the standard model of Moran process with Birth-death updating. A natural direction for future research is to consider related models, such as those with location-dependent fitness [76–79] or those with death-Birth updating [20, 63, 80, 81]. It is known that in terms of fixation probabilities the Birth-death and the death-Birth processes behave quite differently for undirected graphs [37, 45, 82].

## Supporting information

S1 Appendix Supplementary information for fixation times on directed graphs. Contains formal proofs of the claims made in the main text.
(PDF)
