# Supplementary material for: Fixation times on directed graphs
Source: PLoS Comput Biol. 2024 Jul 18;20(7):e1012299. doi: 10.1371/journal.pcbi.1012299 (PMC11288448; doi:10.1371/journal.pcbi.1012299)
Supplement: S1 Appendix — Contains formal proofs of the claims made in the main text. (PDF) [file pcbi.1012299.s001.pdf]

Supplementary Information: Fixation times on directed graphs

David A. Brewster[a], Martin A. Nowak[b,c], and Josef Tkadlec[b,d]

[a]John A. Paulson School of Engineering and Applied Sciences, Harvard University, Cambridge, MA 02138, USA
[b]Department of Mathematics, Harvard University, Cambridge, MA 02138, USA
[c]Department of Organismic and Evolutionary Biology, Harvard University, Cambridge MA 02138, USA
[d]Computer Science Institute, Charles University, Prague, Czech Republic

This is a supplementary information to the manuscript *Fixation times on directed graphs*. It contains formal proofs of the theorems listed in the main text. In Section 1 we formally introduce the model and recall results that we will use in our proofs. In the following sections we prove Theorems 1 to 5 from the main text.

1 Preliminaries

1.1 Moran process on a graph

A *graph* G is a tuple (V, E) where V is a set of *vertices* and E is a set of *edges* between vertices. Unless otherwise specified, graphs are directed, unweighted, strongly connected, do not have self-loops, and do not have multiple edges between vertices. The *outdegree* of a vertex v, denoted $\deg^+(v)$, is the number of outgoing edges, and its *indegree*, denoted $\deg^-(v)$, is the number of incoming edges. When the outdegree equals the indegree, we call it the *degree* and denote it $\deg(v)$. Given a graph, its population size is $N := |V|$. To emphasize the population size of a graph G, we sometimes refer to it as G_N. To model the evolutionary dynamics we consider the Moran Birth-death processes on graphs. In this process, each vertex has an associated *type* at every time step: it is either a *resident* or a *mutant*. Each type has an associated reproductive fitness: residents have fitness 1, mutants have fitness $r \geqslant 1$.

The *Moran Birth-death process* is defined as follows: At each time step we

1. Pick a random vertex $u \in V$ proportional to its type's reproductive fitness.
2. Pick an outgoing edge of u uniformly at random; denote its endpoint $v \in V$.
3. Update the type of v to be the type of u.

Formally, the process is represented as a sequence $X_0, X_1, X_2, \ldots \subseteq V$, where X_0 is the set of vertices that are initially occupied by mutants, and X_t is the set of vertices occupied by mutants after t steps. The set of vertices occupied by mutants is called a *(mutant) configuration*. If G is finite and strongly connected, then with probability one eventually all vertices are occupied by mutants (if $X_t = V$ for some t, we say that the mutants *fixated*) or all vertices are occupied by residents (if $X_t = \emptyset$ for some t, we say that the mutants *went extinct*).

1.2 Fixation probability and time

We define some relevant quantities when studying evolutionary dynamics in a structured population. Each quantity depends on the mutant fitness advantage $r \geqslant 1$; on the underlying population structure, represented by a graph G_N; and on the set X_0 of vertices that are occupied by mutants.

The *fixation probability*, denoted $\mathrm{fp} := \mathrm{fp}_r(G, S)$, is the probability that the mutants eventually take over the population forever starting from a set of mutants occupying vertices $S \subseteq V$. In contrast, the *extinction probability* is the probability that the mutants eventually die out forever. If the graph is strongly connected, fixation or extinction will happen with probability 1 in finitely many expected steps. When the processes reaches fixation or

extinction, we say that the process has *absorbed*. We can also study the time it takes for each of these events to happen: the *expected absorption time* $\mathrm{AT}_r(G,S)$ is the expected amount of time steps until the process absorbs. The *expected fixation time* $\mathrm{T}_r(G,S)$ is the expected amount of time steps conditional on fixation occurring. We also define $\mathrm{T}_r(G)=\max_{S\subseteq V, S\neq\emptyset}\{\mathrm{T}_r(G,S)\}$ as the slowest possible fixation time, across all possible initial conditions. Similarly, we define the *expected extinction time* $\mathrm{ExtT}_r(G,S)$ as the average number of steps over those trajectories that eventually lead to mutant extinction. When the mutant fitness advantage r, the underlying graph G, or the initial condition S are clear from the context we omit it. Further, for a $u\in V$, we sometimes denote $\mathrm{fp}_r(G,\{u\})$ as $\mathrm{fp}_r(G,u)$ for notational ease.

We note that there are two types of steps in the Moran process, namely the *active steps* in which the configuration changes, and the *waiting steps* in which it stays the same. The absorption, fixation, and extinction times defined above count both the active steps and the waiting steps.

Finally, we say that if the expected absorption or fixation time is bounded from above by some polynomial in terms of the population size N then the time is *fast*; otherwise the time is *slow*.

1.3 Forward bias lemma

In the rest of this section, we define the notion of a forward bias and present a lemma that proves useful in deriving Theorems 1 and 2.

Given a strongly connected directed graph $G_N=(V,E)$, a mutant fitness advantage $r\geqslant 1$, and a nonempty set $S\subsetneq V$ of nodes currently occupied by mutants, we define the *up-probability* $p_r^+(S)$ as the probability that, in a single step of the Moran process, the number of mutants increases. In other words, $p_r^+(S)$ is the probability that a mutant is selected for reproduction and its offspring replaces a resident neighbor. Likewise, we define the *down-probability* $p_r^-(S)$ as the probability that the number of mutants decreases. Similarly to above, $p_r^-(S)$ is the probability that a resident is selected for reproduction and its offspring replaces a mutant neighbor. Since the graph G_N is strongly connected and $S\notin\{\emptyset,V\}$ then both $p_r^+(S)$ and $p_r^-(S)$ are non-zero. In that case, we define the *bias at* S as the ratio $\gamma_r(S)=p_r^+(S)/p_r^-(S)$.

Lemma 1. *Let $G_N=(V,E)$ be a strongly connected directed graph with N nodes, $S\subsetneq V$ the nonempty set of nodes initially occupied by mutants, and $r\geqslant 1$ the mutant fitness advantage. Suppose that there exists a real number $f>1$ such that for any nonempty subset $U\subsetneq V$ of nodes we have $\gamma_r(U)\geqslant f$. Then*

1. $\mathrm{fp}_r(G_N,S)\geqslant 1-1/f$,
2. $\mathrm{AT}_r(G_N,S)\leqslant \frac{f+1}{f-1}\cdot N^3$,
3. $\mathrm{T}_r(G_N,S)\leqslant \frac{\mathrm{AT}_r(G_N,S)}{\mathrm{fp}_r(G_N,S)}\leqslant \frac{f(f+1)}{(f-1)^2}\cdot N^3$.

Further, for any $\varepsilon\in(0,1)$ and for parameters r, G_N, and S, the amount of time until absorption given the process fixates is at most $\frac{f(f+1)}{(f-1)^2}\cdot\frac{N^3}{\varepsilon}$ with probability at least $1-\varepsilon$.

To prove Lemma 1 we need the following form of Markov's inequality.

Lemma 2 (Conditional Markov's inequality)**.** *Let X be an almost surely nonnegative random variable. Let $a>0$ and let $\mathcal{E}$ be an event. Then*

$$\Pr[X>a\mid\mathcal{E}]\leqslant\mathbb{E}\left[X/a\mid\mathcal{E}\right]. \tag{1}$$

Proof. Similar to a common proof of Markov's inequality, notice $a\cdot\mathbb{1}_{\{X>a\}}\cdot\mathbb{1}_{\{\mathcal{E}\}}\leqslant X\cdot\mathbb{1}_{\{\mathcal{E}\}}$. Taking the expectation of both sides yields the result. □

We proceed to prove Lemma 1.

Proof of Lemma 1. The key idea is to project the process to a one-dimensional random walk by tracking the number of nodes occupied by mutants. Formally, the random walk is a Markov chain W with states $s_0,s_1,\dots,s_N$ (where state s_k corresponds to those mutant configurations with precisely k mutants), and with transition probabilities $\Pr[s_k\to s_{k+1}]=\frac{f_k}{f_k+1}$, $\Pr[s_k\to s_{k-1}]=\frac{1}{f_k+1}$, where $f_k=\min\{\gamma_r(S)\colon |S|=k\}$ is the smallest forward bias among all the mutant configurations with precisely k mutants. For $k=0,\dots,N$, let p_k be the probability that

W starting at s_k eventually reaches s_N (as opposed to reaching s_0). Note that the random walk W models only the active steps of the Moran process (that is, the steps in which the mutant configuration changes). Moreover, at each configuration it always assumes the lowest possible forward bias. Let $i = |S|$ be the number of nodes initially occupied by mutants. Thus, we have $\mathrm{fp}_r(G_N, S) \geqslant p_i$.

First, we prove Item 1. A standard formula for one-dimensional Markov chains (see e.g. [1, Section 6.2] immediately yields the desired

$$p_i \geqslant p_1 = \frac{1}{1+\sum_{j=1}^{N-1}\prod_{k=1}^{j}\frac{1}{f_k}} \geqslant \frac{1}{\sum_{j=0}^{N-1}(1/f)^j} \geqslant \frac{1}{\sum_{j=0}^{\infty}(1/f)^j} = 1-1/f.$$

Next, we prove Item 2. We deal with the active steps and the waiting steps separately.

Regarding the waiting steps, consider any nonempty mutant configuration $S \subsetneq V$ with $1 \leqslant k \leqslant N-1$ mutants and let $F = k\cdot r + (N-k)\cdot 1 \leqslant r\cdot N$ be the total fitness of the population. Since G_N is strongly connected, there is at least one edge going from a mutant node to a resident node. Since $r \geqslant 1$, the probability $p_a(S)$ that the next step is active satisfies $p_a(S) \geqslant \frac{r}{F}\cdot\frac{1}{N-1} \geqslant \frac{1}{N}\cdot\frac{1}{N} = 1/N^2$. Thus, at any configuration, the expected number of steps until an active step occurs is at most N^2. Therefore, in order to get an upper bound on the absorption time (which includes both the active and the waiting steps), it suffices to count only the active steps and then multiply the result by N^2.

To count the active steps, consider the corresponding random walk W. Given $1 \leqslant k \leqslant N-1$, let x_k be the expected number of times the state s_k is visited in W. We will prove that $x_k \leqslant \frac{f+1}{f-1}$ for each $1 \leqslant k \leqslant N-1$.

To that end, consider a walk W currently at s_k. With probability $\frac{f}{f+1}$ it next moves to s_{k+1}. Once in s_{k+1}, by Item 1 the walk reaches s_N before reaching s_k with probability at least $p_1 \geqslant 1-1/f$. Thus, any time the walk is at s_k, with probability at least $\frac{f}{f+1}\cdot\frac{f-1}{f} = \frac{f-1}{f+1}$ it never comes back. Therefore, $x_k \leqslant 1/\frac{f-1}{f+1} = \frac{f+1}{f-1}$. This is true for any of the $N-1$ states $s_1,\dots,s_{N-1}$, so the expected number of active steps is at most $\frac{f+1}{f-1}\cdot N$ and the expected number of all steps (including the waiting steps) is at most $\mathrm{AT}_r(G_N,S) \leqslant N^2\cdot\left(\frac{f+1}{f-1}\cdot N\right) = \frac{f+1}{f-1}\cdot N^3$.

Finally, we prove Item 3. By linearity of expectation we have

$$\mathrm{AT}_r(G_N,S) = \mathrm{T}_r(G_N,S)\cdot\mathrm{fp}_r(G_N,S) + \mathrm{ExtT}_r(G_N,S)\cdot(1-\mathrm{fp}_r(G_N,S)),$$

where $\mathrm{ExtT}_r(G_N,S)$ is the conditional extinction time, that is, the average length of those stochastic trajectories that terminate with the mutation going extinct. Applying a trivial bound $\mathrm{ExtT}_r(G_N,S) \geqslant 0$ and Item 1, we get

$$\mathrm{AT}_r(G_N,S) \geqslant \mathrm{T}_r(G_N,S)\cdot\mathrm{fp}_r(G_N,S) \geqslant \mathrm{T}_r(G_n,S)\cdot(1-1/f). \tag{2}$$

Putting this together with Item 2 gives the desired

$$\mathrm{T}_r(G_n,S) \leqslant \frac{f}{f-1}\cdot\mathrm{AT}_r(G_N,S) \leqslant \frac{f(f+1)}{(f-1)^2}\cdot N^3.$$

For the last statement of the lemma, we apply Lemma 2 to the time a trajectory takes to absorb as X, the event the process fixates as $\mathcal{E}$, and $a = \varepsilon^{-1}\cdot\mathbb{E}[X \mid \mathcal{E}]$ using Item 3.

□

2 Fixation always occurs quickly when selection advantage is strong enough

In this section we prove Theorem 1 from the main text. That is, we show that if the mutant fitness advantage is large enough then the process terminates fast, regardless of the underlying spatial structure.

Theorem 1. *Let G_N be a strongly connected graph on N nodes. Suppose that $r \geqslant N^2$. Then $\mathrm{AT}_r(G_N) \leqslant 2\cdot N^3$ and $\mathrm{T}_r(G_N) \leqslant 3\cdot N^3$.*

Proof. Suppose mutants currently occupy a nonempty set $S \subsetneq V$ of nodes. Let $k = |S|$ and denote by $F = k\cdot r + (N-k)\cdot 1$ the total fitness of the population.

Since G_N is strongly connected, there is at least one mutant node u with a resident neighbor. We claim that if u is selected for reproduction, then it replaces a resident with probability at least $1/k$. We distinguish two cases based on the outdegree d of u.

1. If $d \leqslant k$ then the claimed probability is at least $\frac{1}{d} \geqslant \frac{1}{k}$.

2. If $d \geqslant k$ then u must have at least $d-(k-1)$ resident neighbors (since there are at most $k-1$ other mutant nodes altogether). Thus the claimed probability is at least $\frac{d-k+1}{d} \geqslant \frac{1}{k}$, where the inequality is equivalent with $(d-k)(k-1) \geqslant 0$ which holds trivially.

Altogether, node u gets selected with probability at least r/F, thus we have:

$$p_r^+(S) \geqslant \frac{r}{F} \cdot \frac{1}{k}.$$

On the other hand, since there are $N-k$ residents and each, when selected for reproduction, replaces a mutant with probability at most 1, we have

$$p_r^-(S) \leqslant \frac{N-k}{F}.$$

Combining those two bounds, we get

$$\gamma_r(S) \geqslant \frac{r}{k(N-k)} \geqslant \frac{r}{N^2/4} \geqslant 4,$$

where we have used an AM-GM inequality for k and $N-k$. Thus, Lemma 1 applies with $f=4$ and we get the desired $\mathrm{AT}_r(G_N) \leqslant \frac{5}{3}N^3 < 2N^3$ and $\mathrm{T}_r(G_N) \leqslant \frac{20}{9}N^3 < 3N^3$. □

3 Fixation occurs quickly on Eulerian graphs

In this section we prove Theorem 2 from the main text. That is, we show that if a graph is Eulerian with degrees sandwiched between δ and Δ then the time is fast, provided that the mutant fitness advantage satisfies $r > \Delta/\delta$. Recall that a graph is *Eulerian* if $\deg^-(v) = \deg^+(v)$ holds for each $v \in V$.

First, we point out one useful property of such graphs. Let $S \subseteq V$. Let $E^+(S)$ be the set of the edges whose starting vertex is in S. Let $E^-(S)$ be the set of the edges whose ending vertex is in S. Let m_S^+ be the number edges outgoing from S and incoming to $V \setminus S$. Similarly, let m_S^- be the number edges outgoing from $V \setminus S$ and incoming to S.

Lemma 3. *A graph $G=(V,E)$ is Eulerian if and only if $m_S^+ = m_S^-$ for every $S \subseteq V$.*

Proof. Suppose G is Eulerian and let $S \subseteq V$. We have that

$$\begin{aligned} m_S^+ &= |E^+(S)| - \#\{u \to v \in E \mid u \in S, v \in S\} \quad \text{and} \\ m_S^- &= |E^-(S)| - \#\{u \to v \in E \mid u \in S, v \in S\}. \end{aligned}$$

Noticing that

$$|E^+(S)| = \sum_{u \in S} \deg^+(u) = \sum_{u \in S} \deg^-(u) = |E^-(S)|$$

since G is Eulerian completes one direction of the proof.

For the other direction, suppose we know that $m^+(S) = m^-(S)$ for each $S \subseteq V$. Then for every $u \in V$ we know $\deg^-(u) = m_{\{u\}}^- = m_{\{u\}}^+ = \deg^+(u)$, so G is Eulerian. □

Theorem 2. *Let G_N be a strongly connected Eulerian graph on N nodes with smallest degree δ and largest degree Δ. Suppose that $r \geqslant \frac{\Delta}{\delta} \cdot (1+\varepsilon)$ for some $\varepsilon > 0$. Then $\mathrm{AT}_r(G_N) \leqslant \frac{2+\varepsilon}{\varepsilon} \cdot N^3$ and $\mathrm{T}_r(G_N) \leqslant \frac{(1+\varepsilon)(2+\varepsilon)}{\varepsilon^2} \cdot N^3$.*

Proof. Suppose mutants currently occupy a nonempty set $S \subsetneq V$ of nodes. Let $E^+ = \{(u,v) \in E \mid u \in S, v \notin S\}|$ be the set of those edges that go from a mutant to a resident. Likewise, let $E^- = \{(u,v) \in E \mid u \notin S, v \in S\}$ be the set of those edges that go from a resident to a mutant. By Lemma 3 we know that the two sets E^+ and E^- have the same size, denote it by s. Denoting by $F = |S| \cdot r + (N-|S|) \cdot 1$ the total fitness of the population, we have

$$p_r^+(S) \geqslant \sum_{(u,v) \in E^+} \frac{r}{F} \cdot \frac{1}{\deg(u)} \geqslant s \cdot \frac{r}{F\Delta}$$

and

$$p_r^-(S) \leqslant \sum_{(u,v)\in E^-} \frac{1}{F} \cdot \frac{1}{\deg(u)} \leqslant s \cdot \frac{1}{F\delta}.$$

Thus

$$\gamma_r(S) \geqslant \frac{r\delta}{\Delta} = 1 + \varepsilon,$$

hence Lemma 1 applies with $f = 1 + \varepsilon$. The claims follow by straightforward algebra. □

4 Fixation can occur slightly faster for small selective advantages

In this section we prove Theorem 3 from the main text. That is, we bound the fixation time on any graph G_N with any mutant fitness advantage $r \geqslant 1$ in terms of the minimum fixation probability $\mathrm{fp}_{\min} := \min_{S\subseteq V, S\neq\emptyset} \mathrm{fp}_{r=1}(G, S)$. The proof has two ingredients. As our first ingredient, we bound the fixation time in the neutral regime ($r = 1$). To that end, we first recall a standard lemma.

Lemma 4 (Corollary 26 of [2], martingale upper additive drift)**.** *Let $Z_0, Z_1, Z_2, \ldots$ be random variables over $[\alpha, \beta] \subseteq \mathbb{R}$, and let $\tau := \inf\{t \geqslant 0 \mid Z_t \in \{\alpha, \beta\}\}$. Furthermore, suppose that,*

1. *$\mathbb{E}[Z_{t+1} \mid Z_0, \ldots, Z_t] = Z_t$ for all $t < \tau$, and*
2. *there is some $\delta > 0$ such that $\mathrm{Var}[Z_{t+1}|Z_0, \ldots, Z_t] \geqslant \delta$ for all $t < \tau$.*

Then

$$\mathbb{E}[\tau \mid Z_0] \leqslant \frac{(Z_0 - \alpha)(\beta - Z_0)}{\delta}. \tag{3}$$

Lemma 4 allows us to bound the absorption time and the fixation time in the neutral regime ($r = 1$).

Lemma 5 (Bounding absorption time and fixation time when $r = 1$)**.** *Let $G = (V, E)$ be strongly connected. Then*

$$\mathrm{AT}_{r=1}(G) \leqslant \frac{N^4}{4\mathrm{fp}_{\min}^2} \quad \textit{and} \quad \mathrm{T}_{r=1}(G) \leqslant \frac{N^4}{4\mathrm{fp}_{\min}^3}.$$

Proof. Note that for any nonempty set $S \subsetneq V$ of mutants, the probability of making an active step is at least $1/N^2$. Indeed, since G is strongly connected, there is a mutant node u with a resident out-neighbor v. Node u is selected for reproduction with probability $r/(r \cdot |S| + (N - |S|)) \geqslant 1/N$, and the offspring replaces v with probability $1/\deg^+(u) > 1/N$. Moreover, since in the neutral regime ($r = 1$) the fixation probability is additive over the set of nodes occupied by mutants, if such an active step happens and node v becomes a mutant, then the fixation probability increases by $\mathrm{fp}_{r=1}(G, \{v\}) \geqslant \mathrm{fp}_{\min}$.

Let $(X_t)_{t\geqslant 0}$ be the mutant configuration after t steps of the Moran process. We aim to apply Lemma 4, where $Z_t = \mathrm{fp}(X_t)$, $\alpha = 0$, and $\beta = 1$. To do that, we bound the conditional variance $\mathrm{Var}[Z_{t+1} \mid Z_0, \ldots, Z_t]$ from below as follows:

$$\begin{aligned}
\mathrm{Var}[Z_{t+1} \mid Z_0, \ldots, Z_t] &= \mathbb{E}\left[(Z_{t+1} - \mathbb{E}[Z_{t+1} \mid Z_0, \ldots, Z_t])^2 \mid Z_0, \ldots, Z_t\right] && (4)\\
&= \mathbb{E}\left[(Z_{t+1} - Z_t)^2 \mid Z_0, \ldots, Z_t\right] && (5)\\
&\geqslant \left(\mathbb{E}\left[|Z_{t+1} - Z_t| \mid Z_0, \ldots, Z_t\right]\right)^2 && (6)\\
&\geqslant \left(\frac{1}{N^2} \cdot \mathrm{fp}_{\min}\right)^2, && (7)
\end{aligned}$$

where in the respective steps we used the definition of conditional variance, the fact that fixation probability does not change in expectation in one step of the process, Jensen's inequality for a convex function $f(x) = x^2$, and the observation that with probability at least $1/N^2$ the fixation probability changes by at least $\mathrm{fp}_{\min}$.

Applying Lemma 4, we find that

$$\mathrm{AT}_{r=1}(G, S) \leqslant \left(\frac{N^2}{\mathrm{fp}_{\min}}\right)^2 \cdot \mathrm{fp}(S)(1 - \mathrm{fp}(S)) \leqslant \frac{N^4}{4\mathrm{fp}_{\min}^2}, \tag{8}$$

for any $S \subseteq V$ where in the last step we used an inequality $x(1-x) \leqslant 1/4$ that holds for any $x \in (0,1)$.

Finally, we turn the obtained bound on absorption time into a bound on fixation time. Let $S \subseteq V$ and denote by $\mathrm{ExtT}_{r=1}(G,S)$ the extinction time starting from S. Then

$$\mathrm{AT}_{r=1}(G,S) = \mathrm{fp}_{r=1}(G,S)\cdot \mathrm{T}_{r=1}(G,S) + (1-\mathrm{fp}_{r=1}(G,S))\cdot \mathrm{ExtT}_{r=1}(G,S) \geqslant \mathrm{fp}_{\min}\cdot \mathrm{T}_{r=1}(G,S),$$

thus taking the maximum over $S \subseteq V$ we obtain $\mathrm{T}_{r=1}(G) \leqslant \mathrm{AT}_{r=1}(G)/\mathrm{fp}_{\min}$. □

As our second ingredient, we relate the fixation time in the neutral regime ($r = 1$) and the fixation time when $r > 1$.

Lemma 6. *Let $G = (V,E)$ be strongly connected, $r \geqslant 1$, and let $\mathrm{fp}_{\min} := \min_{S\subseteq V, S\neq\emptyset} \mathrm{fp}_{r=1}(G,S)$. Then $\mathrm{T}_r(G) \leqslant \frac{4r}{\mathrm{fp}_{\min}}\cdot \mathrm{T}_{r=1}(G)$.*

Proof. Consider the process M_1 with $r = 1$ and the process M_r with $r \geqslant 1$. In M_1, there exist fixating trajectories with probability mass at least $\mathrm{fp}_{\min}$. They fixate on average in at most $T_1 = \mathrm{T}_{r=1}(G)$ steps. Let A_1 be a random variable for the absorption time of a trajectory drawn from M_1 according to the birth-death updating rule. Let $\mathcal{F}$ be the event that a trajectory drawn from M_1 according to the birth-death updating rule fixates. Thus $T_1 = \mathbb{E}[A_1 \mid \mathcal{F}]$. By applying Lemma 2 with $X = A_1$, $a = 2T_1$, and $\mathcal{E} = \mathcal{F}$,

$$\Pr[A_1 > 2T_1 \mid \mathcal{F}] \leqslant \mathbb{E}[A_1 \mid \mathcal{F}]/(2T_1) = 1/2. \tag{9}$$

So for the complementary event $A_1 \leqslant 2T_1$, we have

$$\Pr[A_1 \leqslant 2T_1 \text{ and } \mathcal{F}] = \Pr[A_1 \leqslant 2T_1 \mid \mathcal{F}]\cdot \Pr[\mathcal{F}] \tag{10}$$
$$\geqslant (1/2)\cdot \mathrm{fp}_{\min}. \tag{11}$$

Thus in M_1 there exist fixating trajectories with total probability mass at least $\mathrm{fp}_{\min}/2$ that fixate in at most $2T_1$ steps each.

Let $\tilde{M}_1$ and $\tilde{M}_r$ denote the continuous-time versions of the processes as described in [3]. Then by Lemma 5 of [3], there is a coupling between the continuous-time versions of the two processes such that if the processes start with the same set of mutants (that is, $\tilde{M}_1[0] = \tilde{M}_r[0]$), then $\tilde{M}_1[t] \subseteq \tilde{M}_r[t]$ for all $t \geqslant 0$. Let $\tilde{\tau}_1$ be one possible mutant set trajectory that fixates for $\tilde{M}_1$. Then the coupling implies that the corresponding trajectory $\tilde{\tau}_r$ for $\tilde{M}_r$ fixates even earlier, when measured in the continuous time.

Next, we transfer this relationship back into the world of discrete-time processes M_1 and M_r. Note that at each moment in time, the total fitness of the population in $\tilde{M}_r$ is at most rN, that is, it is at most r times as large as the total fitness of the population in $\tilde{M}_1$. Thus, reproduction events in $\tilde{M}_r$ occur at a rate that is at most r times larger than the rate at which reproduction events occur in $\tilde{M}_1$. When we move from continuous time to discrete time, we count each reproduction event as lasting 1 unit of time. Thus, any time a trajectory $\tilde{\tau}_1 \in \tilde{M}_1$ gives rise to a trajectory $\tau_1 \in M_1$ with length ℓ, the coupled trajectory $\tilde{\tau}_r \in \tilde{M}_r$ gives rise to a trajectory $\tau_r \in M_r$ with length at most $r\ell$.

Because in M_1 there exist fixating trajectories with total probability mass at least $\mathrm{fp}_{\min}/2$ that fixate in at most $2T_1$ steps each, in M_r there exist fixating trajectories with total probability mass $\mathrm{fp}_{\min}/2$ that fixate in at most $2rT_1$ steps each. Now imagine we run M_r for stages of $2rT_1$ steps each. Within each stage, the process fixates with probability at least $\mathrm{fp}_{\min}/2$ so in expectation we observe fixation after at most $2/\mathrm{fp}_{\min}$ stages. In total, this gives the desired

$$\mathrm{T}_r(G) \leqslant \frac{2}{\mathrm{fp}_{\min}}\cdot 2r\cdot T_1 = \frac{4r}{\mathrm{fp}_{\min}}\cdot T_1(G).$$ □

It remains to combine the two ingredients.

Theorem 3. *Let G_N be a strongly connected graph on N vertices and let $r \geqslant 1$. Then $\mathrm{T}_r(G_N) \leqslant \frac{N^6}{\mathrm{fp}_{\min}^4}$.*

Proof. We distinguish two cases. If $r \geqslant N^2$ then Theorem 1 implies that $\mathrm{T}_r(G_N) \leqslant 3N^3$ which is stronger than the claimed bound. So suppose $r < N^2$. Then Lemmas 5 and 6 yield

$$\mathrm{T}_r(G_N) \leqslant \frac{4r}{\mathrm{fp}_{\min}}\cdot \mathrm{T}_{r=1}(G_N) \leqslant \frac{4r}{\mathrm{fp}_{\min}}\cdot \frac{N^4}{4\mathrm{fp}_{\min}^3} \leqslant \frac{N^6}{\mathrm{fp}_{\min}^4}.$$ □

5 Balanced graphs (and others) fixate quickly

Lemma 7. *Let $G = (V, E)$ be strongly connected. Suppose there exist $|V|$ numbers $\{x_v \mid v \in V\}$ that satisfy*

$$x_v \cdot \sum_{u:u\to v\in E} \frac{1}{\deg^+(u)} = \frac{1}{\deg^+(v)} \cdot \sum_{w:v\to w\in E} x_w \tag{12}$$

for each $v \in V$. In addition, suppose $\sum_{v\in V} x_v = 1$. Then $x_v = \mathrm{fp}(\{v\})$.

Proof. Suppose $r = 1$ and denote $N = |V|$. Then the fixation probability is additive, that is, $\mathrm{fp}(S) = \sum_{v\in S} \mathrm{fp}(\{v\})$ for every $S \subseteq V$ [4]. Thus, the list of 2^N fixation probabilities $\mathrm{fp}(S)$ for $S \subseteq V$ is determined by the list of N fixation probabilities $\mathrm{fp}(\{v\})$ for $v \in V$. The fixation probabilities $\mathrm{fp}(\{v\})$ are the unique solutions to the linear system

$$\mathrm{fp}(\{v\}) = \frac{1}{N} \sum_{v\to w\in E} \frac{1}{\deg^+(v)} \cdot \mathrm{fp}(\{v,w\}) + \frac{1}{N} \sum_{u\to v\in E} \frac{1}{\deg^+(u)} \cdot \mathrm{fp}(\emptyset) + \left(1 - \frac{1}{N} - \frac{1}{N} \sum_{u\to v\in E} \frac{1}{\deg^+(u)}\right) \cdot \mathrm{fp}(\{v\}), \tag{13}$$

where the first term on the right-hand side corresponds to the mutant reproducing, the second term corresponds to the mutant being replaced, and the third term corresponds to a resident replacing a resident. Using $\mathrm{fp}(\emptyset) = 0$ and $\mathrm{fp}(\{v,w\}) = \mathrm{fp}(\{v\}) + \mathrm{fp}(\{w\})$ we obtain

$$\mathrm{fp}(\{v\}) \cdot \sum_{u:u\to v\in E} \frac{1}{\deg^+(u)} = \frac{1}{\deg^+(v)} \cdot \sum_{w:v\to w\in E} \mathrm{fp}(\{w\}) \tag{14}$$

which is precisely the system satisfied by $\{x_v \mid v \in V\}$.

□

Definition 1. *A strongly connected graph $G = (V, E)$ is* balanced *if and only if*

$$\frac{1}{\deg^-(v)} \cdot \sum_{u:u\to v\in E} \frac{1}{\deg^+(u)} = \frac{1}{\deg^+(v)} \cdot \sum_{w:v\to w\in E} \frac{1}{\deg^-(w)}. \tag{15}$$

Theorem 4. *Let G_N be a balanced strongly connected graph. Then:*

1. $\mathrm{fp}_{r=1}(G_N, u) = \frac{1/\deg^-(u)}{\sum_{v\in V} 1/\deg^-(v)} \geqslant 1/N^2$ *for any node u.*
2. $\mathrm{T}_r(G_N) \leqslant N^{14}$ *for any $r \geqslant 1$.*

Proof. The equality in Item 1 follows from Lemma 7 and the bound follows from the fact that the numerator is at least $1/N$ and the denominator is at most N. Item 2 follows immediately from Item 1 and from Theorem 3. □

In the rest of this section we verify that the undirected graphs, carousels, books, metafunnels, and superstars are all balanced. Thus, the fixation probability under neutral drift ($r = 1$) starting from node v is inversely proportional to $\deg^-(v)$. Moreover, we provide an explicit formula for fixation probability on Megastars under neutral drift. This gives an upper bound on the fixation time for any $r \geqslant 1$ by Theorem 3. We use the notation $\mathrm{fp}(\{v\}) \propto p_v$ to mean that $\mathrm{fp}(\{v\}) = p_v / \sum_{w\in V} p_w$.;

Recall that a graph is *undirected* if all edges are two-way, that is, if $u \to v \in E$ then $v \to u \in E$ as well. A weakly connected undirected graph is also strongly connected since each edge can be traversed in either direction.

Claim 1. *Suppose $G = (V, E)$ is undirected. Then $\mathrm{fp}(\{v\}) \propto 1/\deg(v)$.*

Proof. Checking by substituting,

$$\frac{1}{\deg(v)} \sum_{v\to w\in E} \frac{1}{\deg(w)} = \frac{1}{\deg(v)} \sum_{u\to v\in E} \frac{1}{\deg(u)} \tag{16}$$

$$\iff \frac{1}{\deg(v)} \sum_{\substack{v\to w\in E\\ w\to v\in E}} \frac{1}{\deg(w)} = \frac{1}{\deg(v)} \sum_{\substack{u\to v\in E\\ v\to u\in E}} \frac{1}{\deg(u)} \tag{17}$$

$$\iff \frac{1}{\deg(v)} = \frac{1}{\deg(v)} \tag{18}$$

which is always true. □

For the following claims, we omit the proofs since the technique is similar of that of the proof of claim 1.

Definition 2. *A* carousel *is multipartite graph consisting of a partition of V into sets $S_1, \dots, S_\ell$ such that $u \to v \in E$ if and only if there exists an i such that $u \in S_i$ and $v \in S_{i+1}$. We say that $S_{\ell+1} := S_1$, $S_0 := S_\ell$, $S_{-1} := S_{\ell-1}$, etc.*

Claim 2. *Suppose $G = (V, E)$ is a carousel with sets $S_1, \dots, S_\ell$. Then for $v \in S_i$ we have that* $\mathrm{fp}(\{v\}) \propto |S_{i-1}|^{-1}$.

Proof. We have

$$|S_{i-1}|^{-1} \cdot \sum_{u \to v \in E} \frac{1}{\deg^+(u)} = |S_{i-1}| \cdot |S_i|^{-1} |S_{i-1}|^{-1} = |S_i|^{-1} \tag{19}$$

and

$$\frac{1}{\deg^+(v)} \sum_{v \to w \in E} |S_i|^{-1} = |S_i|^{-1}. \tag{20}$$

□

Definition 3. *An $(s_1, \dots, s_\ell)$-*book *is a graph with vertices b (beginning), e (end), and $p^i_{j_i}$ for $i = 1, \dots, \ell$ and $j_i = 2, \dots, s_{i-1}$. We say that $p^i_1 = p^i_{s_i+1} = b$ and $p^i_{s_i} = e$ for each i. We have $p^i_{j_i} \to p^i_{j_i+1} \in E$ for all $j_i = 1, \dots, s_i$*

Claim 3. *Suppose $G = (V, E)$ is a $(s_1, \dots, s_\ell)$-book. Then*

1. $\mathrm{fp}(\{b\}) \propto \ell$
2. $\mathrm{fp}(\{e\}) \propto 1$
3. $\mathrm{fp}(\{p^i_2\}) \propto \ell$ *for each i*
4. $\mathrm{fp}(\{p^i_j\}) \propto 1$ *for each i and each $j > 1$.*

Proof. This can be easily checked by plugging into the equations of (14). □

Definition 4. *See §1.1.1 of [5] for the definition of a (k, ℓ, m)-*metafunnel.

Claim 4. *Suppose $G = (V, E)$ is a (k, ℓ, m)-metafunnel. Then*

1. $\mathrm{fp}(\{v\}) \propto m^{1-i}$ *for each $v \in V_i$ such that $i \neq 1$*
2. $\mathrm{fp}(\{v\}) \propto \ell$ *for each $v \in V_1$.*

Definition 5. *See §1.1.2 of [5] for the definition of a (k, ℓ, m)-*superstar.

Claim 5. *Suppose $G = (V, E)$ is a (k, ℓ, m)-superstar. Then*

1. $\mathrm{fp}(\{v_{i,j}\}) \propto 1$ *for each i and each $j > 1$*
2. $\mathrm{fp}(\{v_{i,1}\}) \propto 1/m$ *for each i*
3. $\mathrm{fp}(\{v\}) \propto \ell$ *for each $v \in R_i$ for each i*
4. $\mathrm{fp}(\{v^*\}) \propto 1$.

Corollary 1. *Undirected graphs, carousels, books, metafunnels, and superstars are balanced.*

Proof. This follows from Theorem 4, and Claims 1 to 5. □

Definition 6. *See §1.1.3 of [5] for the definition of a (k, ℓ, m)-*megastar.

Claim 6. *Suppose $G = (V, E)$ is a (k, ℓ, m)-megastar. Then*

1. $\mathrm{fp}(\{v^*\}) \propto m$

2. $\mathrm{fp}(\{v\}) \propto \ell m$ *for each* $v \in R_1 \cup \cdots \cup R_\ell$

3. $\mathrm{fp}(\{v\}) \propto m$ *for each* $v \in K_1 \cup \cdots \cup K_\ell$

4. $\mathrm{fp}(\{a_i\}) \propto 1$ *for each* $i \in [\ell]$.

Corollary 2. *A* (k, ℓ, m)*-megastar has* $\mathrm{fp}_{min} \geqslant 1/h(N)$ *for some polynomial* N *so long as*

$$1 \cdot \ell + m \cdot (k\ell) + (\ell m) \cdot (\ell m) + m \leqslant h(N). \tag{21}$$

In particular, so long as k, ℓ, and m are each bounded above by some polynomial in N then $\mathrm{fp}_{\min}$ is bounded below by the inverse of a polynomial.

6 Quickly estimating fixation probabilities with provable confidence

Theorem 5. *For strongly connected* $G = (V, E)$*,* $u \in V$*, and* $r \geqslant 1$*, there is a fully polynomial randomised approximation scheme (FPRAS) for computing* $\mathrm{fp}_r(G, u)$ *if there is some polynomial* h *such that* $\mathrm{fp}_{\min} \geqslant 1/h(N)$*.*

Proof. What follows is a standard technique. We aim to approximate the fixation probability within a multiplicative factor of $\varepsilon > 0$ with probability $1 - \nu$. Let $\nu_1, \nu_2 \geqslant 0$ be constants and let $\nu := \nu_1 + \nu_2$. We first compute $\mathrm{fp}_{\min}$ and then T_1 in poly(N) time by Lemma 7. We run $s = \lceil 2(\ln 2/\nu_1)/(\varepsilon \mathrm{fp}_{\min})^2 \rceil$ independent simulations of the birth-death process on G for at most $t = \lceil T_1 s/\nu_2 \rceil$ steps. If any simulation does not reach absorption in the allocated time, we return some arbitrary number as the fixation probability. Otherwise, take $X_1, \ldots, X_s$ to be random 0-1 indicator variables such that $X_i = 1$ if and only if the simulation reaches fixation. We estimate $\hat{\mathrm{fp}}_r(G, u) := \frac{1}{s} \sum_{i=1}^{s} X_i$ as the fixation probability. Then $\Pr[|\hat{\mathrm{fp}}_r(G, u) - \mathrm{fp}_r(G, u)| > \varepsilon \, \mathrm{fp}_r(G, u)] \leqslant 2 \exp(-\varepsilon^2 s \, \mathrm{fp}_r(G, u)^2/2) \leqslant \nu_1$ by a Chernoff bound. The probability that a simulation does not reach absorption in the allocated amount of time is at most ν_2/s by Markov's inequality. By a union bound, the probability that there is some simulation that does not reach absorption in the allocated time is at most ν_2. Thus the probability of error in this approximation algorithm is at most $\nu_1 + \nu_2 = \nu$. Both s and t are polynomial in N and each step of the birth-death process can be computed in a constant amount of time. □

7 Computer experiments

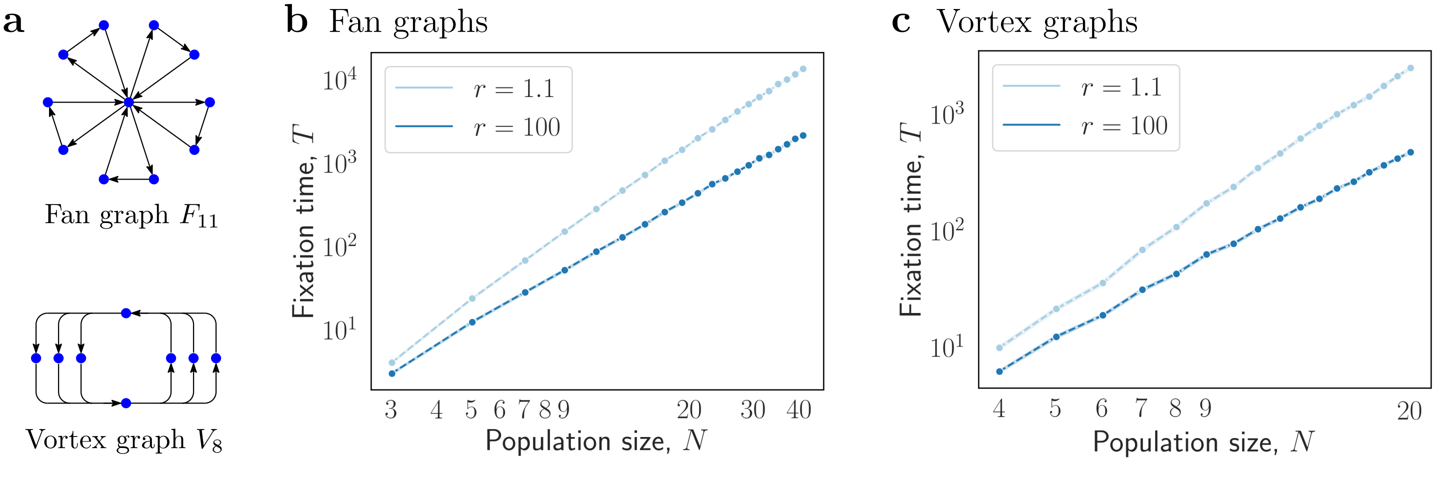

Figure 1: **Fixation time on slow oriented graphs. a,** The Fan graph with k blades has $N = 2k + 1$ nodes and $3k$ one-way edges (here $k = 5$ which yields $N = 11$). The Vortex graph with batch size k has $N = 2k + 2$ nodes and $4k$ edges (here $k = 3$ which yields $N = 8$). **b-c,** For both the Fan graphs and the Vortex graphs the fixation time scales roughly as N^2, both for $r = 1.1$ and $r = 100$. Each data point is an average over 100 simulations. We note that the plots are on a log-log scale.

References

[1] Martin A Nowak. *Evolutionary dynamics: exploring the equations of life*. Harvard University Press, 2006.

[2] Timo Kötzing and Martin S Krejca. First-hitting times under drift. *Theoretical Computer Science*, 796:51–69, 2019.

[3] Josep Díaz, Leslie Ann Goldberg, David Richerby, and Maria Serna. Absorption time of the moran process. *Random Structures & Algorithms*, 49(1):137–159, 2016.

[4] M Broom, C Hadjichrysanthou, J Rychtář, and BT Stadler. Two results on evolutionary processes on general non-directed graphs. *Proceedings of the Royal Society A: Mathematical, Physical and Engineering Sciences*, 466(2121):2795–2798, 2010.

[5] Andreas Galanis, Andreas Göbel, Leslie Ann Goldberg, John Lapinskas, and David Richerby. Amplifiers for the moran process. *Journal of the ACM (JACM)*, 64(1):1–90, 2017.
